# High Incidence of Moderately Reduced Renal Function and Lead Bioaccumulation in Agricultural Workers in Assin South District, Ghana: A Community-Based Case-Control Study

**DOI:** 10.1155/2019/5368427

**Published:** 2019-09-30

**Authors:** Patrick Adu, Eric Kumah Forkuo, Abubakari Issah, Isaac Owusu Asumadu, Emmanuel Cadman-Sackey, Augustina A. A. Quarshie, Sampson Gyabaa, Richard K. D. Ephraim

**Affiliations:** ^1^Department of Medical Laboratory Science, School of Allied Health Sciences, University of Cape Coast, Cape Coast, Ghana; ^2^Medical Laboratory Unit, Ewim Polyclinic, Cape Coast, Ghana

## Abstract

**Background:**

The quest to enhance agricultural productivity and crop yields has led to increased use of agrochemicals on a global scale. Long-term use of these agrochemicals may be associated with adverse health implications.

**Objective:**

To assess haematological indices, renal function, heavy metal bioaccumulation in farmers and sprayers, and their use of personal protective equipment (PPE).

**Materials and Methods:**

This community-based case-control study was conducted from January 2018 to June 2018 in the Assin South District, Central Region, Ghana. A total of 144 participants were conveniently sampled: 83 agricultural workers (cases) and 61 indigenes with no direct exposure to agrochemicals (controls). Structured questionnaire was used to obtain demographic data as well as agricultural work practices followed by cases. Venous blood samples were drawn from participants and used for estimating full blood count and renal function (serum creatinine (CRE), blood-urea nitrogen (BUN), BUN : CRE ratio, and estimated GFR (eGFR)). Serum lead, arsenic, and cadmium levels were estimated using the Varian AA 240FS atomic spectrometer in an acetylene-air flame.

**Results:**

The median RBC (4.49 vs. 4.92 × 10^12^/L), haemoglobin (12.50 vs. 13.70 g/dL), and platelet (220.00 vs. 268.00) counts were significantly lower in cases. A significantly higher proportion of cases were classified as anaemic or having microcytic cells compared to controls. Also, serum urea (4.08 vs. 3.41; *p*=0.0009), creatinine (108.10 vs. 101.10; *p*=0.0286), and BUN : CRE ratio (19.75 vs. 17.84) were significantly higher in cases. Additionally, 18.1% of cases were classified as having moderately reduced renal function compared to only 6.6% of controls. Moreover, a significantly higher proportion of cases had detectable serum lead (55.6% vs. 16.4%) and arsenic (53.1% vs. 9.8%) levels compared to controls. However, on average, 80% of agricultural workers did not use personal protective equipment (PPE) when applying agrochemicals; 84.3% of used agrochemical containments were discarded near the river/canal.

**Conclusion:**

Neglect of the use of PPE may be predisposing the agrochemical workers and community to lead and arsenic bioaccumulation with a consequent reduced haematological and renal function.

## 1. Introduction

Agrochemicals (pesticides and fertilizers) are used in agricultural practices to safeguard crops from damage by pest and to enhance agricultural productivity. In Ghana, the agricultural sector employs over half of the national labour force and contributes above 20% to the GDP [1]. It is estimated that 87% of farmers in Ghana use chemical pesticides to control pests and diseases [2]. Globally, the use of chemical fertilizers has increased tremendously since the 1960s and has led to massively increased crop production. The challenge with the use of chemical fertilizers is groundwater contamination, accumulation in crops, and long-term effects of heavy metal contamination [3, 4]. Analytical testing of a wide range of fertilizer products by several studies [5–7] shows that inorganic fertilizers and liming materials contain elevated levels of arsenic, cadmium, and lead compared to organic types. In spite of these adverse health implications, the increased agricultural yield associated with the use of these agrochemicals has led to their preference over biological, cultural, and mechanical methods for boosting production [8]. As they are biologically active substances, long-term exposure to these agrochemicals may have both acute and chronic health-related effects. Farmers and those who spray these agrochemicals are exposed to different concentrations and varieties of agrochemicals (organophosphates, organochlorines, etc.), leading to bioaccumulation of these chemicals which can induce different cellular alterations and diseases [9–11]. These toxicities however can be significantly reduced by using personal protective equipment (PPE) and the frugal handling of agrochemicals.

The impact of long-term pesticide exposure on human kidney function remains an area of active research [12–15]. A variety of pesticide classes (organophosphate, organochlorine, carbamate, pyrethroid insecticides, triazine, and chlorophenoxy herbicides) have been shown to cause renal damage and dysfunction in animals [16]. Also, higher levels of organochlorine pesticides were detected in chronic kidney disease (CKD) patients along with a reduced glomerular filtration rate and increased oxidative stress in certain epidemiological studies [17].

Farmers in Ghana spend a life time in this occupation and so stand the risk of adverse health-related changes associated with chronic exposure to agrochemicals. In this case-control study, we sought to compare the haematological and renal functions of farmers directly exposed to agrochemicals to those of people not directly exposed to these agrochemicals to assess the health implications of long-term exposure to the agrochemicals. Additionally, we also sought to evaluate the observance of preventive measures meant to minimize the health hazards of these agrochemicals by farmers and sprayers.

## 2. Materials and Methods

### 2.1. Study Site

This case-control study was carried out at Sibinso in the Assin South District of the Central Region, Ghana. The estimated population of Assin South District using the Central Region population growth rate of 3.1% is 323,156.4, of which an estimated 67.0% are classified as skilled agricultural, forestry, and fishery workers [1].

### 2.2. Study Design

This community-based study targeted all farmers in the Sibinso community. The chief and unit committee of the town organized a durbar for the research team to educate and explain the rationale of this study to the community. After clarifying any questions, only individuals who gave informed consent were recruited for this study. Overall, a total of 144 participants (19–60 years) from the community agreed to be part of this study. Of them, 83 (57.6%) indicated of being farmers or sprayers in the community with more than six months of working experience in the agricultural sector and with direct exposure to agrochemicals (cases). Sixty-one (42.4%) were sampled from the community as healthy individuals who were neither farmers nor sprayers and therefore had no direct exposure to agrochemicals (controls). However, individuals within the community who aided the farmers or the sprayers were excluded as they were deemed to have had a higher chance of direct exposure to agrochemicals. Since pesticides' usage and exposure have been linked to various chronic diseases, individuals who have had neurologic disorders or been diagnosed with kidney diseases, haematological disorders, or any other chronic illness were excluded.

### 2.3. Questionnaire

A well-structured questionnaire was used to obtain sociodemographic data of both cases and controls. Information on agricultural work practices followed by the case subjects (farmers and sprayers) was also obtained.

### 2.4. Blood Sample Collection

After obtaining participants' consent, 6 ml of venous blood sample was drawn from each participant following the standard protocol. 4 ml of the venous blood sample was dispensed into a serum gel separator tube for the clear separation of the serum, which was aliquoted into Eppendorf tubes and stored for later biochemical analysis, while the 2 ml left was dispensed into EDTA (ethylenediaminetetraacetic acid) tubes for haematological analysis.

### 2.5. Haematological Analysis

Full blood count (FBC) parameters for each participant was estimated using Mindray BC-2800 Hematology Analyzer (China) following the manufacturers' protocol after the analyzer was confirmed to have passed quality control samples analyzed.

### 2.6. Biochemical Analysis

Serum urea and creatinine were estimated as markers of kidney function using the Selectra PRO S 13-96 automated clinical biochemistry analyzer (Elitech Group, France) at the Cape Coast Teaching Hospital (CCTH). The BUN : CRE ratio was also estimated as part of the kidney function test for each participant. All experimental procedures were done following standard operating protocols [3, 14, 18]. The glomerular filtration rate (GFR) was estimated using the CKD-EPI creatinine equation taking into consideration gender, age, and serum creatinine levels in accordance with previously published data [19].

### 2.7. Wet Digestion for Heavy Metals

For the estimation of serum lead, arsenic, and cadmium for each participant, aliquoted sera were transported to the Environmental Chemistry Laboratory at Ghana Atomic Energy Commission (GAEC). These heavy metals were selected as they have been shown to be among the most heavy metals with serious health implications [20]. 2 g of the serum sample was transferred into a 100 ml class “A” beaker; 20 ml of conc. HNO_3_ and 2 ml of conc. H_2_O_2_ were added to the sample in a fume chamber. The beaker was covered with a cling film, placed on a hot plate, and digested for 3 h at a temperature of 45°C. After the acid digestion, the mixture was left to cool and then transferred into a 50 ml measuring cylinder; distilled water was added to make a final volume of 20 ml. After that, the whole content was transferred into test tubes and then assayed for the presence of lead (Pb), cadmium (Cd), and arsenic (As) using the Varian AA 240FS atomic absorption spectrometer in an acetylene-air flame. The instrument detection limit for various heavy metals was 0.002 mg/l, 0.001 mg/l, and 0.001 mg/l for cadmium, lead, and arsenic, respectively. Reference standards used for the elements of interest, blanks, and duplicates of samples were digested under the same conditions as the samples. Reference standards used were from Fluka Analytical (Sigma-Aldrich GmbH, Switzerland).

### 2.8. Data Analysis

IBM SPSS Statistics for Windows, Version 22.0 (IBM Corporation, USA), was used for the statistical analysis of data generated. Data were analyzed and presented as percentages and mean ± SD. The D'Agostino–Pearson normality test was used to assess the normality of data. Those that passed or otherwise were analyzed using the independent *T*-test and Mann–Whitney *U* test, respectively. For all statistical calculations, *p* < 0.05 was considered to be significant.

## 3. Results

### 3.1. Community Demographic Information

The demographic information of the individual participants typified that of the Ghanaian farming community. This is evident in having most of the farmers within the age range of 40–49 years in both cases and controls (41.0% cases vs. 32.8% controls); 19–29 years was the least represented group (Table 1). A majority of the participants were male (67.5% cases vs. 68.9% controls), which typifies the community to be a more farming and rural one. While only 3.6% of cases had tertiary education, 6.6% of controls had tertiary education. However, the secondary education level was the predominant educational attainment for both cases and controls. Also, comparable numbers of participants neither smoked (96.4% cases vs. 96.7% controls) nor took alcoholic beverage (60.2% cases vs. 60.7% controls).

### 3.2. Haematological Indices

This study also assessed the haematological parameters of participants (Table 2). The median RBC (4.49 vs. 4.92; *p*=0.0002), haemoglobin (12.5 vs. 13.7 g/dL; *p*=0.0005), and platelet (220 vs. 268) counts were significantly lower in cases compared to those in controls. A significantly higher proportion of cases were anaemic compared to controls (37.3% cases vs. 16.4% controls). Additionally, while 21.7% of cases had microcytosis, 9.8% of controls had microcytosis.

### 3.3. Renal Function Analyses

When renal function of participants was assessed (Table 3), cases had significantly higher mean serum urea (4.08 vs. 3.41) and creatinine (108.1 vs. 101.1) compared to controls. The median blood urea nitrogen-to-creatinine ratio was significantly higher in cases compared to that in controls. A significantly higher proportion of cases had an elevated BUN : CRE ratio compared to controls (48.8% cases vs. 31.1% controls; *p*=0.0094). Also, when the GFR was estimated for participants, while only 25.3% of cases had normal GFR, 41.0% of controls had normal GFR. Additionally, while 18.1% of cases had moderately reduced renal function, 6.6% of controls had moderately reduced renal function.

### 3.4. Heavy Metal Level Evaluation

Heavy metal accumulation in the serum of participants was also assessed (Table 4). While Pb was detectable in the serum of 55.6% of the cases, only 16.4% of controls had detectable Pb in the serum (*p* < 0.0001). Also, while 53.1% of cases had detectable serum levels of As, only 9.8% of controls had detectable As in the serum. Moreover, the median of the serum Pb levels of cases was significantly higher than that of controls (1.180 in cases vs. 0.278 in controls; *p*=0.0003).

### 3.5. Usage of PPE by Agricultural Workers

The implementation of personal protective measures by cases was explored (Table 5). A majority of agricultural workers have not gone through any integrated PEST management training. Although 96.4% of the cases reported never wearing gloves when mixing agrochemicals, 32.5% reported using bare hands to mix agrochemicals. A majority of the agrochemical workers did not apply basic personnel protective measures such as use of goggles (95.2%), nose masks (89.2%), and overall (83.1%). Interestingly, 84.3% of the agrochemical workers reported disposing of containment of agrochemicals near rivers or canals. The agrochemicals used by the farmers were generally classified as class II or III under the WHO categorization based on toxicity (see Supplementary [Supplementary-material supplementary-material-1]).

## 4. Discussion

Our study provides evidence of systematic neglect of the routine use of personal protective equipment by agricultural workers (farmers and agrochemical sprayers) among the study participants. As agrochemicals have been shown to be absorbed through the skin and other mucous membranes, the potential for systemic accumulation of residues of these agrochemicals over a prolonged use by the agricultural workers in our study area remains to be quantified in a well-controlled longitudinal study. Our case-control study demonstrates that agricultural workers had comparatively reduced haematopoietic output and renal function when compared to individuals who reside in the same community and thus share similar social amenities but are not directly exposed to these agrochemicals. Considering that, on average, over 80% of the agricultural workers in our study population did not use any form of basic personal protective equipment such as gloves, goggles, or overcoats during application of these agrochemicals, these findings may be expected.

It is noteworthy that not only do a substantial proportion of these agricultural workers choose to use bare hands to mix and apply the agrochemicals to crops but also discard the containment of these used agrochemicals near rivers or canals, thus exposing the entire community to the hazards of these agrochemicals. Not surprisingly, nearly one-fifth of controls had detectable lead levels (as well as approximately one-tenth had detectable arsenic levels) which increases to over a half of the agricultural workers with detectable lead and arsenic levels in the study population. We speculate that failure of these agricultural workers to properly dispose the containment of used agrochemicals leads to leaching of residuals into the source of drinking water and food that are consumed by indigenes of these communities which might explain the levels of heavy metals detected in our controls. This gross neglect of use of PPE is in spite of the fact that the agrochemicals used by these farmers were classified as class II (moderately hazardous) and class III (slightly hazardous) in terms of toxicity to human health (see Supplementary [Supplementary-material supplementary-material-1]). Low adherence of agricultural workers to the use of PPE when applying agrochemicals has been reported by other studies [21–23]. Regarding the higher proportion of agricultural workers with detectable levels of lead and arsenic, we hypothesize that skin contamination as well as inhalation of agrochemicals dispersed through the air as a result of the limited use of PPE might directly account for these findings. The basis of our hypothesis stems from the fact that most of the agricultural workers stated that their clothes are always wet during application of these agrochemicals. To begin the process of proper environmental management of the situation, it may be an interesting prospect to pilot local farmer association in these communities as membership of such association was found to increase the adoption of safe working practices among farmers in Oman [21, 24, 25].

Although our study was cross-sectional in nature and did not determine the long-term impact of the use of agrochemicals and their indiscriminate disposal on future health outcomes, the findings provide a snapshot of the potential adverse consequences on renal function as approximately one-fifth (18.1%) of the apparently healthy agricultural workers had moderately reduced renal function as quantified by GFR measurements. Epidemiological data have demonstrated increased chronic kidney disease in individuals with higher levels of heavy metals [26, 27]. It was recently shown that simultaneous exposure to glyphosate and heavy metals increased nephrotoxicity in Sri Lanka [28]. Evidence has also been provided linking occupational exposure or drinking underground well water in farming communities as the source of heavy metals and its consequent chronic kidney disease [29, 30]. It should be noted that renal pathology is not the only adverse health outcome linked to the use of these agrochemicals. A recent meta-analysis estimated a 41% increased risk of non-Hodgkin's lymphoma in individuals with high cumulative exposure to glyphosate-based herbicides [31]. Again, a large population-based cohort study in the US found increased cardiovascular-related mortality due to low levels of lead exposure [32]. As some of the agrochemicals used by our study participants were glyphosate-based (see Supplementary [Supplementary-material supplementary-material-1]), a longitudinal study is warranted to elucidate the full impact of exposure to these agrochemicals throughout the span of life. For example, as some studies found considerable levels of glyphosate or its metabolite, AMPA in urine and breast milk [33, 34], and paraquat in the serum of Thai women [35], it will be interesting to systematically track the impact of these on pregnancy outcomes. Our study however did not assess the biolevels of these agrochemicals to establish the causal link to the reported reduced renal function. In line with the indiscriminate handling of the agrochemicals demonstrated by agricultural workers in the study population, it will be interesting to measure the biolevels of these agrochemicals in the agricultural workers, and particularly in children in our study population as they play mostly in the soil.

The use of agrochemicals has gained grounds in the Ghanaian agricultural sector because of aggressive advertisement as well as government subsidies on these agrochemicals to boost agricultural outputs. However, our study raises questions that need to be addressed through conscientious scholarly research. For example, there is a need for quantification of how pregnancy outcomes may be impacted through these indiscriminate disposals of agrochemical containment in these agricultural-based communities. Also, another question is how do these emerging agricultural practices affect growth and development of children or the mortality rates in these communities. These are some key questions that will be addressed through future studies.

## 5. Conclusions

The agricultural workers in the study area are oblivious of the adverse health implications of bioaccumulation of agrochemical residues and heavy metals. Educational strategies aimed at heightening the importance of using PPE to minimize bioaccumulation of these agrochemicals should be pursued.

## Figures and Tables

**Table 1:** Demographic details of participants.

Parameter	Cases *N* (%)	Controls *N* (%)	*p* value
Age (years)			**0.0152**
19–29	5 (6.0)	13 (21.3)	
30–39	13 (15.7)	10 (16.4)	
40–49	34 (41.0)	20 (32.8)	
50–60	31 (37.3)	18 (29.5)	
Gender			0.8787
Male	56 (67.5)	42 (68.9)	
Female	27 (32.5)	19 (31.1)	
Education			0.2233
Primary	12 (14.5)	14 (23.0)	
Secondary	53 (63.9)	31 (50.8)	
Tertiary	3 (3.6)	4 (6.6)	
Vocational	7 (8.4)	4 (6.6)	
No formal education	8 (9.6)	8 (13.1)	
Smoking			
No	80 (96.4)	59 (96.7)	
Yes	3 (3.6)	2 (3.3)	
Alcohol intake			
No	50 (60.2)	37 (60.7)	
Yes	33 (39.8)	24 (39.3)	

Boldface indicates that significantly higher proportion of the controls belonged to the 19‐29 age group compared to the cases.

**Table 2:** Haematological profile of study participants.

Parameter	Cases	Controls	*p* value
RBC (×10^12^/L)	4.49	4.92	**0.0002**
Haemoglobin (g/dl)	12.50	13.70	**0.0005**
Total WBC (×10^9^/L)	4.90	5.00	0.1730
Platelet (×10^9^/L)	220.00	268.00	**0.0085**
^*∗*^Anaemia classification			
Anaemia	31 (37.3)	10 (16.4)	**0.0007**
Normal	52 (62.7)	51 (83.6)	
MCV			0.0564
<80 fL (microcytosis)	18 (21.7)	6 (9.8)	
80–98 fL (normocytic cells)	63 (75.9)	53 (86.9)	
>98 fL (macrocytosis)	2 (2.4)	2 (3.3)	

Boldface indicates that RBC count, haemoglobin concentration, and platelet counts in cases were significantly lower in cases compared to respective values in controls; for anaemia classification, boldface indicates that significantly higher proportion of cases were anaemic compared to controls.

**Table 3:** Renal function of participants.

Parameter	Cases (*N* = 83)	Controls (*N* = 61)	*p* value
Urea (mmol/L)	4.08 ± 0.13	3.41 ± 0.15	0.0009
Creatinine (*μ*mol/L)	108.10 ± 2.25	101.10 ± 2.09	0.0286
BUN : CRE ratio	19.75 (7.92)	17.84 (7.04)	0.0212^†^
BUN : CRE ratio classification			0.0094
1–20	42 (51.2)	42 (68.9)	
>20	40 (48.8)	19 (31.1)	
eGFR classification			0.0106
G1 (≥90)	21 (25.3)	25 (41.0)	
G2 (60–89)	47 (56.6)	32 (52.5)	
G3a (45–59)	12 (14.5)	4 (6.6)	
G3b (30–44)	3 (3.6)	0 (0.0)	

Parameters with superscript “†” were compared using median as data were nonparametric; all other parameters were compared using mean (±SD) as data were parametric; BUN : CRE means blood urea nitrogen-to-creatinine.

**Table 4:** Heavy metal levels in the serum of participants.

	Cases (*N* = 81)	Controls (*N* = 61)	*p* value
Lead (Pb)			<0.0001^z^
Detectable	45 (55.6)	10 (16.4)	
Undetectable	36 (44.4)	51 (83.6)	
Arsenic (As)			
Detectable	43 (53.1)	6 (9.8)	<0.0001^z^
Undetectable	38 (46.9)	55 (90.2)	
Cadmium (Cd)			
Detectable	1 (1.2)	0 (0)	
Undetectable	80 (99.8)	61 (100)	
Serum levels of heavy metals (mg/L)			
Pb	1.180	0.2782	0.0003^†^
As	0.09023	0.09383	0.8964^†^

^z^Proportions compared using the chi-square test; ^†^median compared using the Mann–Whitney *U* test.

**Table 5:** Safety practices of cases.

Questions related to safety practices of cases		*N* (%)
Have you undertaken any integrated PEST management training?		
	No	43 (51.8)
	Yes	40 (48.2)
How do you mix agrochemicals?		
	By machine	13 (15.7)
	Using bare hands	27 (32.5)
	Using rod	38 (45.8)
	Do not mix agrochemicals	5 (6.0)
Do you wear gloves when preparing/applying agrochemical?		
	No	80 (96.4)
	Yes	3 (3.6)
Do you wear goggles when preparing/applying agrochemical?		
	No	79 (95.2)
	Yes	4 (4.8)
Do you wear nose masks when preparing/applying agrochemical?		
	No	74 (89.2)
	Yes	9 (10.8)
Do you wear overall when preparing/applying agrochemical?		
	No	69 (83.1)
	Yes	14 (16.9)
Do you wear closed shoes when preparing/applying agrochemical?		
	No	42 (47.0)
	Yes	41 (49.4)
Do you break to eat when spraying/applying agrochemicals?		
	No	55 (66.3)
	Yes	28 (33.7)
Do you wash clothes used during spraying of agrochemicals with other household clothes?		
	No	62 (74.7)
	Yes	21 (25.3)
How do you dispose of containment of agrochemical?		
	Buried/burned	12 (14.5)
	Discarded near the river/field/canal	70 (84.3)
	Used at home	1 (1.2)

## Data Availability

The data used to support the findings of this study are available from the corresponding author upon reasonable request.

## Supplementary Materials

Supplementary Materials Supplementary Data S1: list of agrochemicals that are applied to farm products and weeds by the agricultural workers in the Assin South District of Ghana. The list was compiled from responses of the agricultural workers to specific questions in a questionnaire that was administered as part of the data collection tools in this study.
