# Supplementary material for: High Incidence of Moderately Reduced Renal Function and Lead Bioaccumulation in Agricultural Workers in Assin South District, Ghana: A Community-Based Case-Control Study
Source: Int J Nephrol. 2019 Sep 30;2019:5368427. doi: 10.1155/2019/5368427 (PMC6791189; doi:10.1155/2019/5368427)
Supplement: Supplementary Materials — Supplementary Data S1: list of agrochemicals that are applied to farm products and weeds by the agricultural workers in the Assin South District of Ghana. The list was compiled from responses of the agricultural workers to specific questions in a questionnaire that was administered as part of the data collection tools in this study. [file 5368427.f1.docx]

**Supplementary data S1: List of agrochemicals that are applied to farm products and weeds by the agricultural workers at the Assin-south district of Ghana.** The list was compiled from responses of the agricultural workers to specific questions in a questionnaire that was administered as part of the data collection tools in the study.

| **Common name** | **Active Ingredient** | **Chemical type** | **WHO class of toxicity** |
| --- | --- | --- | --- |
| **Adwumawura** | Glyphosate | Organophosphate | III |
| Roundoff | Glyphosate | Organophosphate | III |
| Sunphosate | Glyphosate | Organophosphate | III |
| Kalach | Glyphosate | Organophosphate | III |
| Gramoquat | Paraquat dichloride | Bipyridyl | II |
| Gramazone | Paraquat dichloride | Bipyridyl | II |
| Buffalo | acetamiprid | Neonicotinoids | III |
| **Akate Master** | Bifenthrin | Pyrethroid | II |
| **Akatesuro** | Diazinon | Organophosphate | II |
| Confidor | Imidacloprid | Neonicotinoids | II |
| Paraquat | Paraquat | Bipyridyl | II |
|  |  |  |  |
| Cocofeed | NPK (ratio 0-30-20) | fertilizer |  |
| Ammonia | Ammonium nitrate | fertilizer |  |
| **Assasewura** | PK, magnesium and Sulphur (ratio 0-22-18) | fertilizer |  |
| Urea | NPK (ratio 46-0-0) | fertilizer |  |

N (Nitrogen), P (phosphorus), K (potassium); names in bold print are trade names used in Ghana to market these products.
